# Recovery in Mental Health: A Synthesis of the Perspectives of Experts by Experience, Recovery‐Oriented Professionals and Family Caregivers in Relation to the CHIME and SPICE Frameworks

**DOI:** 10.1111/hex.70815

**Published:** 2026-08-19

**Authors:** Estefania Guerrero, Maite Barrios, Hernán María Sampietro, Juana Gómez‐Benito, Georgina Guilera

**Affiliations:** ^1^ Department of Social Psychology and Quantitative Psychology University of Barcelona Barcelona Catalonia Spain; ^2^ Institute of Neurosciences University of Barcelona Barcelona Catalonia Spain; ^3^ ActivaMent Catalunya Associació Barcelona Catalonia Spain

**Keywords:** CHIME, experts by experience, family, mental health recovery, professionals, recovery frameworks, SPICE

## Abstract

**Background:**

Recovery in mental health is a complex and contested concept shaped by stakeholder perspectives. This paper aimed to (1) analyse convergence between experts by experience and recovery‐oriented professionals regarding the meaning of recovery and progress recognition and facilitators and obstacles, and to examine how the resulting statements align with the CHIME (i.e., Connectedness, Hope, Identity, Meaning in life, and Empowerment) and SPICE (i.e., Social recovery, Prosperity, Individual recovery and Clinical recovery experience) frameworks; and (2) investigate how family caregivers' supportive actions converge with these perspectives and align with both frameworks.

**Methods:**

We conducted secondary qualitative analyses of three international e‐Delphi studies. In Study 1, statements from experts by experience and recovery‐oriented professionals were compared to identify convergence and unique contributions and were then examined in relation to CHIME and SPICE frameworks. In Study 2, family caregivers' supportive actions were compared with Study 1 statements concerning the meaning of recovery and progress recognition to examine convergence and were then examined in relation to both frameworks. The Standards for Reporting Qualitative Research were followed.

**Results:**

Experts by experience and recovery‐oriented professionals converged on recovery as involving rebuilding a meaningful life, agency, empowerment, self‐determination, autonomy, positive identity, connection and safety. Experts by experience emphasised advocacy for rights, financial security, everyday well‐being, social support and critical perspectives on diagnosis and treatment. Shared facilitators and obstacles included living environments, coercion, unmet needs, social support, discrimination, social exclusion, self‐ and social stigma, and holistic approaches to care. Professionals highlighted occupational opportunities, autonomy, self‐awareness, peer support and community‐based care, whereas experts by experience emphasised ethical practice, rights and violence‐free care. Statements concerning the meaning of recovery and progress recognition were most frequently classified under Empowerment in CHIME and Individual recovery in SPICE; facilitators and obstacles were most frequently classified under Empowerment and Connectedness in CHIME and under Social recovery and Prosperity in SPICE. Most supportive actions showed convergence and aligned with both frameworks; those with no direct convergence concerned caregivers' capacities and well‐being.

**Conclusions:**

Despite differences across stakeholder perspectives, the convergences identified provide a foundation for advancing recovery‐oriented mental health care, future research and service development.

**Patient and Public Involvement Statement:**

People with lived experience were actively involved throughout all stages of the research. A mental health service user association (ActivaMent Catalunya Associació) was involved in every phase of the three primary studies from which data for this article were drawn, as well as in the preparation of this article. Specifically, experts by experience participated in defining the research questions and outcomes, designing and managing the two studies described in this paper, and analysing and interpreting the data. The person who contributed directly to this article is included as a co‐author.

## 1 Introduction

Recovery in mental health is a complex and contested concept shaped by the perspectives of diverse stakeholders, including experts by experience, professionals and family members [1, 2, 3, 4, 5, 6, 7]. Traditionally, recovery has primarily been defined in terms of symptom reduction and functional improvement [8, 9, 10]. However, service user and survivor movements have advanced a personal recovery approach, highlighting self‐determination, empowerment and the right to a meaningful life irrespective of ongoing symptoms [1, 11, 12]. These perspectives coexist in practice, often generating tensions within mental health systems and constraining the implementation of genuinely person‐centred, rights‐based services [13, 14].

This conceptual evolution has also generated debate regarding the nature of recovery itself. Personal recovery has been conceptualised both as a non‐linear journey and in terms of a destination or valued outcomes [15]. Framing recovery as a journey avoids a binary distinction between being recovered and not recovered but may make it more difficult to identify when meaningful goals have been achieved, whereas an outcome‐oriented framing makes such goals more visible but may suggest a fixed endpoint [16]. Rather than viewing these framings as mutually exclusive, they may be understood as complementary: indicators and valued outcomes can mark progress within an ongoing, context‐dependent process of personal recovery without implying that recovery has been completed.

The CHIME framework synthesises five core *processes* of personal recovery—Connectedness, Hope, Identity, Meaning in life, and Empowerment [7]—and is widely used to inform policy and practice [17]. More recently, the SPICE framework has sought to broaden prevailing recovery conceptualisations by describing four *dimensions* in which recovery may manifest: Social recovery (both externally and internally derived), Prosperity (i.e., political and economic recovery), Individual recovery (i.e., normalcy, knowledge, appearance and hygiene, individual responsibility and identity), and Clinical recovery experience (i.e., diagnosis and treatment) [5]. By embedding political, economic and socio‐environmental factors, SPICE aligns with current global policy priorities (i.e., emphasising social inclusion, equity and structural determinants of mental health) [18].

Although CHIME has accumulated empirical support as a useful organising framework for personal recovery across culturally diverse groups [19, 20, 21] and different diagnostic perspectives [22, 23, 24], it has also been criticised for its limited capacity to explicitly account for socio‐structural and material conditions shaping recovery, including economic, political and environmental factors [25]. SPICE is a comparatively recent framework and, beyond its original derivation from a systematic review [5], it is unclear to what extent it functions as a robust explanatory framework for recovery across populations and contexts, or whether its structural dimensions add incremental value for conceptualisation, measurement and intervention design.

Recovery‐oriented services are increasingly informed by international guidelines [26] that promote user empowerment through self‐determination, rights and trauma‐informed care, while also seeking to address power imbalances within mental health systems. However, implementing recovery‐oriented care remains challenging due to persistent definitional ambiguity and limited training [27, 28], as well as divergent perspectives among stakeholders—professionals, family members and service users—on the recovery process [5, 28, 29]. To date, limited research has systematically examined how these stakeholder perspectives converge and diverge from one another, and how they align with established recovery frameworks.

To address this gap, the present paper draws on data from three international e‐Delphi studies conducted with experts by experience, recovery‐oriented professionals and family caregivers, which identified group‐level perspectives for each stakeholder group. Through two sequential studies, we sought:

1. To analyse how the perspectives of experts by experience and recovery‐oriented professionals converge on key elements of recovery, and how they align with the CHIME and SPICE frameworks.
2. To investigate how the supportive actions proposed by family caregivers for fostering recovery converge with the perspectives of experts by experience and recovery‐oriented professionals, and how they align with the CHIME and SPICE frameworks.

## 2 Methods

The two studies involved qualitative analysis of consensus‐based outputs, with analytical procedures tailored to their respective research questions. The Standards for Reporting Qualitative Research (SRQR) were followed [30].

### 2.1 Data Source

#### 2.1.1 Study 1

This study compared consensus statements identified in two previous Delphi studies. In both studies, participants responded to four open‐ended questions addressing: (1) the meaning of recovery in mental health, (2) how recovery progress is recognised, (3) factors that facilitate the recovery process and (4) factors that hinder the recovery process. Their parallel structure and equivalent consensus‐based outputs enabled comparison of their conceptual content at the statement level.

The Delphi study with experts by experience included 101 participants, whereas the study with recovery‐oriented professionals included 78 participants (see Table 1 for participant details and Table S1 for recruitment details). The Delphi study with experts by experience [1] resulted in 26 statements on the meaning of recovery, 31 on recognising recovery progress, 8 facilitators and 12 obstacles. The Delphi study with recovery‐oriented professionals [2] yielded 14 statements on the meaning of recovery, 20 on recognising recovery progress, 17 facilitators and 23 obstacles. These consensus statements are publicly accessible [1, 2].

**Table 1:** Sociodemographic characteristics of experts by experience, recovery‐oriented professionals, and family caregivers.

Variables	Participants
Experts by experience (*n* = 101)	
Age (years) *M* (SD)	47.8 (10.8)
Gender *n* (%)	
Female	52 (51.5)
Male	44 (43.6)
Non‐binary	1 (1.0)
Not reported	4 (4.0)
Involvement in an association of service users and/or survivors of psychiatry association *n* (%)	
Yes	81 (80.2)
No	20 (19.8)
Geographical region of residence (by continent) *n* (%)	
Africa	6 (5.9)
America	11 (10.9)
Asia	7 (6.9)
Europe	72 (71.3)
Oceania	5 (5.0)
Recovery‐oriented professionals (*n* = 78)	
Gender *n* (%)	
Female	46 (59.0)
Male	32 (41.0)
Profession *n* (%)	
Lawyer	5 (6.4)
Nurse	13 (16.7)
Occupational therapist	4 (5.1)
Psychiatrist	11 (14.1)
Psychologist	21 (26.9)
Public health specialist	3 (3.9)
Social worker	11 (14.1)
Other	10 (12.8)
Years of professional experience *n* (%)	
2–4 years	2 (2.6)
5–7 years	7 (9.0)
More than 8 years	69 (88.4)
Geographical region of employment (by continent) *n* (%)	
Africa	6 (7.7)
America	10 (12.8)
Asia	9 (11.5)
Europe	49 (62.8)
Oceania	4 (5.1)
Family caregivers (*n* = 53)	
Age (years) *M* (SD)	60.4 (12.9)
Gender *n* (%)	
Female	18 (34.0)
Male	35 (66.0)
Involvement in an association of families of people with mental health problems, *n* (%)	
Yes	46 (86.8)
No	7 (13.2)
Living together with the service user *n* (%)	
Yes	23 (43.4)
No	30 (56.6)
Relationship to the service user *n* (%)	
Sibling	15 (28.3)
Parent	14 (26.4)
Spouse	4 (7.5)
Offspring (adult child)	17 (32.1)
Other	3 (5.7)
Geographical region of residence (by continent) *n* (%)	
Africa	1 (1.9)
America	7 (13.2)
Asia	10 (18.9)
Europe	35 (66.0)

#### 2.1.2 Study 2

Data were drawn from a previous Delphi study involving family caregivers in which consensus was reached on 47 *supportive actions* for recovery [3]. These *supportive actions* are publicly available in the original publication. A total of 53 family caregivers participated in the study (see Table 1 for participant details and Table S1 for recruitment details).

Unlike the Delphi studies included in Study 1, which focused on the meaning of recovery, progress recognition, facilitators, and obstacles, the Delphi study with family caregivers explored actions that could support the recovery of a mental health service user. Consequently, the 47 *supportive actions* were not considered directly equivalent to the consensus statements identified in Study 1. Instead, they were analysed separately, following the Study 1 analyses, as actions intended to support recovery.

### 2.2 Analytical Approach

#### 2.2.1 Study 1

The analysis was conducted in three sequential steps. First, before comparing the perspectives of experts by experience and recovery‐oriented professionals, the statements were prepared separately for each dataset. Statements derived from Questions 1 and 2 (i.e., the meaning of recovery and progress recognition) that reflected the same underlying concept were synthesised into a single integrative statement while preserving their original conceptual content. Statements without a closely related counterpart within the same dataset were retained individually. The same procedure was applied separately to statements derived from Questions 3 and 4 (i.e., facilitators and obstacles). This resulted in two integrated lists for each stakeholder group: one comprising statements concerning the *meaning of recovery and progress recognition*, and the other comprising statements concerning *facilitators and obstacles*. The statements contained in these integrated lists constituted the units of analysis for Study 1.

Second, the integrated lists prepared separately for experts by experience and recovery‐oriented professionals were compared to identify convergence and unique contributions. The initial comparison was conducted by the first author. Statements from the two stakeholder perspectives were matched when they expressed the same or closely related underlying meanings. Statements for which no conceptually corresponding statement was identified from the other stakeholder group were considered unique contributions. This procedure was applied separately to statements concerning the *meaning of recovery and progress recognition* and to statements concerning *facilitators and obstacles*. The proposed convergences and unique contributions were reviewed by the research team and revised through discussion until consensus was reached.

Third, the statements resulting from this comparison were examined in relation to the predefined CHIME processes and SPICE dimensions. Multiple coding was permitted; therefore, a single statement could be aligned with more than one CHIME process and/or SPICE dimension. Statements concerning the *meaning of recovery and progress recognition* were classified under the CHIME processes and SPICE dimensions that corresponded to their conceptual content. Statements concerning *facilitators and obstacles* were classified under the CHIME processes and SPICE dimensions they were considered most likely to influence within the recovery process.

A 30% sample of statements was selected for independent human coding and comparison with the AI‐assisted classifications. For the human coding, two researchers independently coded the statements according to the CHIME processes and SPICE dimensions. Their coding decisions were compared, and any disagreements were discussed with a third member of the research team until consensus was reached. For the AI‐assisted coding, ChatGPT (version 4o, web interface) generated provisional CHIME and SPICE classifications for all statements using systematically developed prompts specifying the analytical task and the predefined CHIME processes and SPICE dimensions [31]. An illustrative prompt is provided in Table S2 (column a). Agreement between the AI‐assisted classifications and the consensus classifications derived from human coding was assessed using Cohen's kappa [32]. Based on Altman's criteria [33], agreement between AI‐assisted and human coding was very good for statements concerning the *meaning of recovery and progress recognition* (*κ* = 0.95) and for statements concerning *facilitators and obstacles* (*κ* = 0.83).

Following this agreement assessment, the research team reviewed all AI‐generated classifications and their accompanying justifications, assessing their conceptual appropriateness, consistency with the consensus classifications derived from human coding, and preservation of the original meaning. Any discrepancies or uncertainties were discussed until consensus was reached. The research team retained responsibility for all final analytical decisions.

#### 2.2.2 Study 2

The analysis was conducted in two sequential steps. First, each *supportive action* proposed by family caregivers was compared with the statements on the *meaning of recovery and progress recognition* identified in Study 1 to examine conceptual convergence. These Study 1 statements served as conceptual points of reference.

Convergence was established when a *supportive action* was considered to directly promote or facilitate the recovery experience represented by a specific Study 1 statement. For example, the *supportive action* ‘eliminating our own stigma towards the user’ was considered to converge with the statement ‘overcoming self‐stigma’. Although the latter refers to the service user's internal experience, reducing stigma within the social environment was interpreted as creating relational conditions that support the development of a positive identity and, consequently, the recovery process.

Second, the *supportive actions* were examined in relation to the predefined CHIME processes and SPICE dimensions. When a *supportive action* converged conceptually with a statement identified in Study 1, it received the same CHIME and SPICE classifications as that statement. *Supportive actions* that showed no direct convergence with any Study 1 statement were examined directly in relation to the CHIME processes and SPICE dimensions they were considered most likely to support.

ChatGPT (version 4o, web interface) used a structured prompt to generate provisional proposals concerning conceptual convergence and classifications into CHIME processes and SPICE dimensions for all *supportive actions*. The exact prompt is provided in Table S2 (column b). Given the high level of human–AI agreement observed in Study 1, an AI‐assisted coding procedure was used in Study 2. All proposed convergences and classifications into CHIME processes and SPICE dimensions, together with their justifications, were reviewed by the research team for conceptual appropriateness, consistency with the analytical decisions established in Study 1 and preservation of the original meaning of the *supportive actions*. Any disagreements or uncertainties were discussed until consensus was reached.

Consistent with recommendations for combining digital and manual qualitative analysis methods [34], ChatGPT outputs across both studies were treated as provisional analytical proposals requiring human validation. The AI‐assisted procedure complemented rather than replaced qualitative judgement, and the research team retained responsibility for all final analytical decisions and the interpretation of the findings. The researchers brought complementary expertise in clinical psychology, methodology and lived experience, providing diverse perspectives that enriched the interpretation of the findings.

Frequencies and percentages were used across Studies 1 and 2 to summarise: (i) the number of statements showing convergence or identified as unique contributions in Study 1, (ii) *supportive actions* showing direct convergence or identified as having no direct convergence in Study 2 and (iii) statements or *supportive actions* classified under CHIME processes and SPICE dimensions, as appropriate. Percentages were calculated based on the total number of statements or *supportive actions* in the corresponding dataset. In Study 1, convergence and unique contributions were mutually exclusive within each comparison; in Study 2, direct convergence and no direct convergence were also mutually exclusive. In contrast, multiple coding was permitted within each framework because a statement or *supportive action* could align with more than one process or dimension. Consequently, percentages for classifications within each framework may sum to more than 100%. These frequencies and percentages are descriptive only; they should not be interpreted as measures of the relative importance of CHIME processes or SPICE dimensions, or of framework performance. Statements or *supportive actions* were not classified under a given framework when they did not align with any of its processes or dimensions.

**Figure 1:**
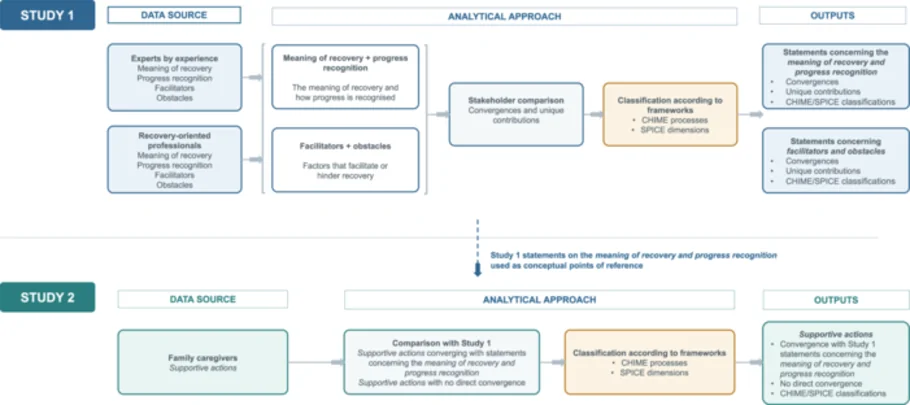
Overview of the data sources and analytical sequence across Study 1 and Study 2.

Figure 1 provides an overview of the data sources and analytical approach used across Studies 1 and 2.

## 3 Results

### 3.1 Meaning of Recovery and Progress Recognition

Of the 33 statements concerning the *meaning of recovery and progress recognition*, 21 (63.6*%*) reflected convergence among experts by experience and recovery‐oriented professionals. These statements clustered around interconnected aspects describing recovery as rebuilding life, growth, self‐awareness, coping, self‐determination, agency, empowerment, autonomy, recognition as a subject of rights, quality of life, meaning in life, leisure, overcoming self‐stigma, feeling good about oneself, hope, connection, and feeling accepted and safe. The remaining 12 statements (36.4*%*) were all unique to experts by experience and included aspects related to relational recovery (e.g., social support and interpersonal relationships), everyday well‐being (e.g., healthy lifestyles), socio‐economic security (e.g., having basic financial needs covered), rights‐based recovery (e.g. defending collective rights and legal capacity) and a critical reorientation away from the dominant biomedical model.

Statements were most commonly classified under the CHIME processes of Empowerment (36.4*%*; e.g., agency and autonomy) and Meaning in life (27.3*%*; e.g., purpose and a fulfilling life), followed by Connectedness (18.2*%*; e.g., belonging and social support) and Identity (9.1*%*; e.g., positive identity and overcoming self‐stigma). Only one statement was classified under Hope (3.0*%*; e.g., hope for the future). Five statements (15.2*%*) were not classified under any CHIME process. One convergent statement concerned safety, while four unique contributions from experts by experience concerned collective rights, healthy lifestyles, financial security and a critical reorientation away from the dominant biomedical approach to diagnosis and treatment.

All statements were classified under at least one SPICE dimension, primarily Individual recovery (66.7*%*; e.g., coping, agency, and meaningful living), followed by Social recovery (18.2*%*; e.g., connection and social support) and Prosperity (12.1*%*; e.g., rights, financial security, and safety). Only one statement was classified under Clinical recovery experience (3.0*%*), reflecting a critical position regarding diagnosis and treatment [e.g., Moving away from the dominant biomedical model approach (i.e. reducing emphasis on diagnosis, clinical input, involuntary detention, and forced treatment, while overcoming the adverse effects of psychiatric drugs and treatments)]. Comparing the classification of statements across the CHIME and SPICE frameworks illustrates that the same content sometimes aligned with complementary aspects of the two frameworks. For example, being recognised as a subject with rights aligned with Empowerment in CHIME and with Prosperity in SPICE.

**Figure 2:**
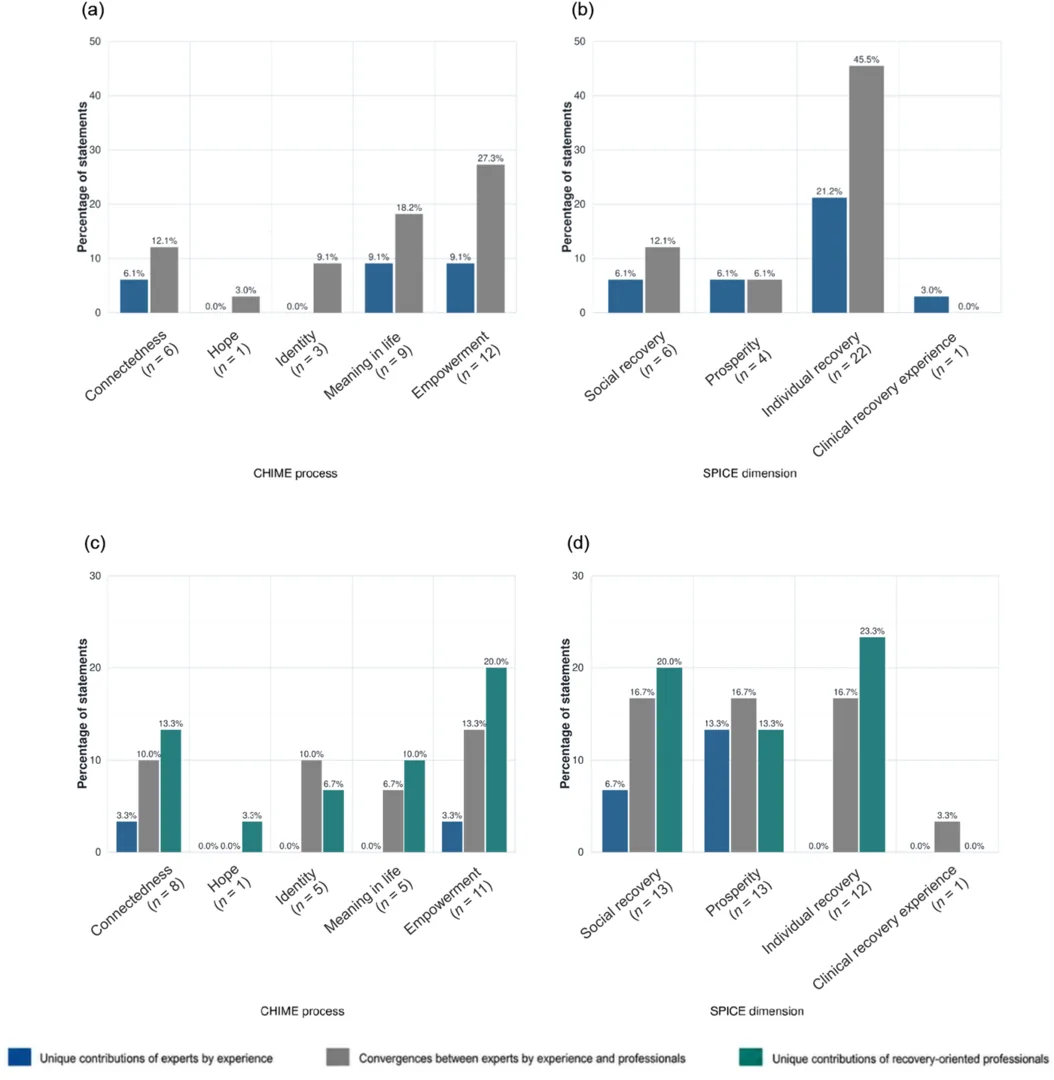
Convergences between and unique contributions of experts by experience and recovery‐oriented professionals in relation to the 33 statements on the *meaning of recovery and progress recognition* and the 30 statements concerning *facilitators and obstacles*, showing how the statements aligned with (a, c) CHIME and (b, d) SPICE. *Note*. Panels (a) and (b) present the CHIME and SPICE classifications, respectively, of statements on the *meaning of recovery and progress recognition*, whereas panels (c) and (d) present the CHIME and SPICE classifications, respectively, of statements concerning *facilitators and obstacles*. Each statement could align with one or more CHIME processes or SPICE dimensions. Five statements on the *meaning of recovery and progress recognition* (15.2*%*) were not classified under any CHIME process; four were unique contributions from experts by experience, and one reflected convergence between stakeholder groups. Eight statements concerning *facilitators and obstacles* (26.7*%*) were not classified under any CHIME process; three were unique contributions from experts by experience, one was a unique contribution from recovery‐oriented professionals, and four reflected convergences between stakeholder groups. Percentages for the CHIME and SPICE classifications, together with the percentage of statements not classified under any CHIME process, may sum to more than 100% because a statement could align with more than one CHIME process or SPICE dimension.

Figure 2 shows convergences between and unique contributions of experts by experience and recovery‐oriented professionals in relation to the 33 statements concerning the *meaning of recovery and progress recognition*, showing how the statements aligned with CHIME and SPICE. Table S3 provides the full set of *meaning of recovery and progress recognition* statements, indicating whether they represent convergent or unique contributions, and their classifications within CHIME and SPICE.

### 3.2 Facilitators and Obstacles

Of the 30 statements concerning *facilitators and obstacles*, 14 (46.7*%*) reflected convergence among experts by experience and recovery‐oriented professionals, 4 (13.3*%*) were unique to experts by experience and 12 (40.0*%*) were unique to professionals. Convergences pointed to living environment, coercion, needs, significant support, discrimination, social exclusion, self‐ and social stigma, and a holistic approach to treatment. Experts by experience highlighted ethical practice, rights and violence‐free care, while professionals emphasised occupational opportunities, autonomy, self‐awareness, peer support and community‐based care.

Statements addressing *facilitators and obstacles* were most commonly classified under the CHIME processes of Empowerment (36.7*%*; e.g., self‐determination and autonomy) and Connectedness (26.7*%*; e.g., significant and peer support), followed by Meaning in life (16.7*%*; e.g., occupational and meaningful activities) and Identity (16.7*%*; e.g., overcoming stigma). Only one statement was classified under Hope (3.3*%*; e.g., gaining hope through professionals and the community). Eight statements (26.7*%*) were not classified under any CHIME process. Four convergent statements concerned the availability of basic services, safe environments, financial security and protection against discrimination; three statements unique to experts by experience concerned inclusive societies, respect for rights and freedom from psychiatric violence; and one statement unique to recovery‐oriented professionals concerned access to adequate and affordable care services.

Regarding SPICE, statements concerning *facilitators and obstacles* were most frequently classified under Social recovery (43.3*%*; e.g., supportive environments and community‐based care) and Prosperity (43.3*%*; e.g., rights, service access, financial security, and safety), followed by Individual recovery (40.0*%*; e.g., psychological resources and self‐awareness). Only one statement was classified under Clinical recovery experience (3.3*%*), emphasising a holistic treatment approach rather than an exclusive emphasis on symptom reduction (e.g., Treatment approach is holistic, not only focused on symptoms). As with the findings for the *meaning of recovery and progress recognition*, CHIME and SPICE sometimes captured complementary aspects of the same statement. For example, support from significant others was classified under Connectedness in CHIME and Social recovery in SPICE.

Figure 2 displays convergences between and unique contributions of experts by experience and recovery‐oriented professionals in relation to the 30 statements concerning *facilitators and obstacles* to recovery, together with their classifications within CHIME and SPICE. Table S4 provides the full set of statements concerning *facilitators and obstacles*, indicating whether they represent convergent or unique contributions, and their classifications within CHIME and SPICE.

### 3.3 Supportive Actions

Of the 47 *supportive actions*, 43 (91.5*%*) directly converged with statements on the *meaning of recovery and progress recognition*, while 4 (8.5*%*) showed no direct convergence and concerned caregivers' own capacities, well‐being and access to support.

Regarding CHIME, 38 *supportive actions* (80.9*%*) were classified under one or more processes, most commonly Empowerment (38.3*%*; e.g., respecting decisions), followed by Connectedness (19.1*%*; e.g., maintaining supportive relationships), Meaning in life (19.1*%*; e.g., encouraging meaningful activities), Identity (10.6*%*; e.g., reducing stigma), and Hope (4.3*%*; e.g., fostering optimism). Nine *supportive actions* (19.1*%*) were not classified under any CHIME process—these concerned facilitating access to services, monitoring treatment adverse effects, seeking professional advice, encouraging health‐promoting behaviours, ensuring financial resources, and four actions focused on caregivers' own capacities and well‐being: managing one's own emotions and concerns, learning about mental health, participating in a family association and practising self‐care.

In relation to SPICE, all 47 *supportive actions* were classified under at least one dimension, primarily Individual recovery (61.7*%*; e.g., practical and relational support), followed by Social recovery (21.3*%*; e.g., promoting inclusion) and Prosperity (10.6*%*; e.g., facilitating access to services and financial resources). Fewer *supportive actions* were classified under Clinical recovery experience (6.4*%*; e.g., monitoring medication effects).

Consistent with Study 1, CHIME and SPICE captured overlapping yet complementary aspects of *supportive actions*. For example, respecting decisions was classified under Empowerment in CHIME and Individual recovery in SPICE, whereas learning about mental health was classified under Clinical recovery experience in SPICE, with no direct correspondence to any CHIME process.

**Figure 3:**
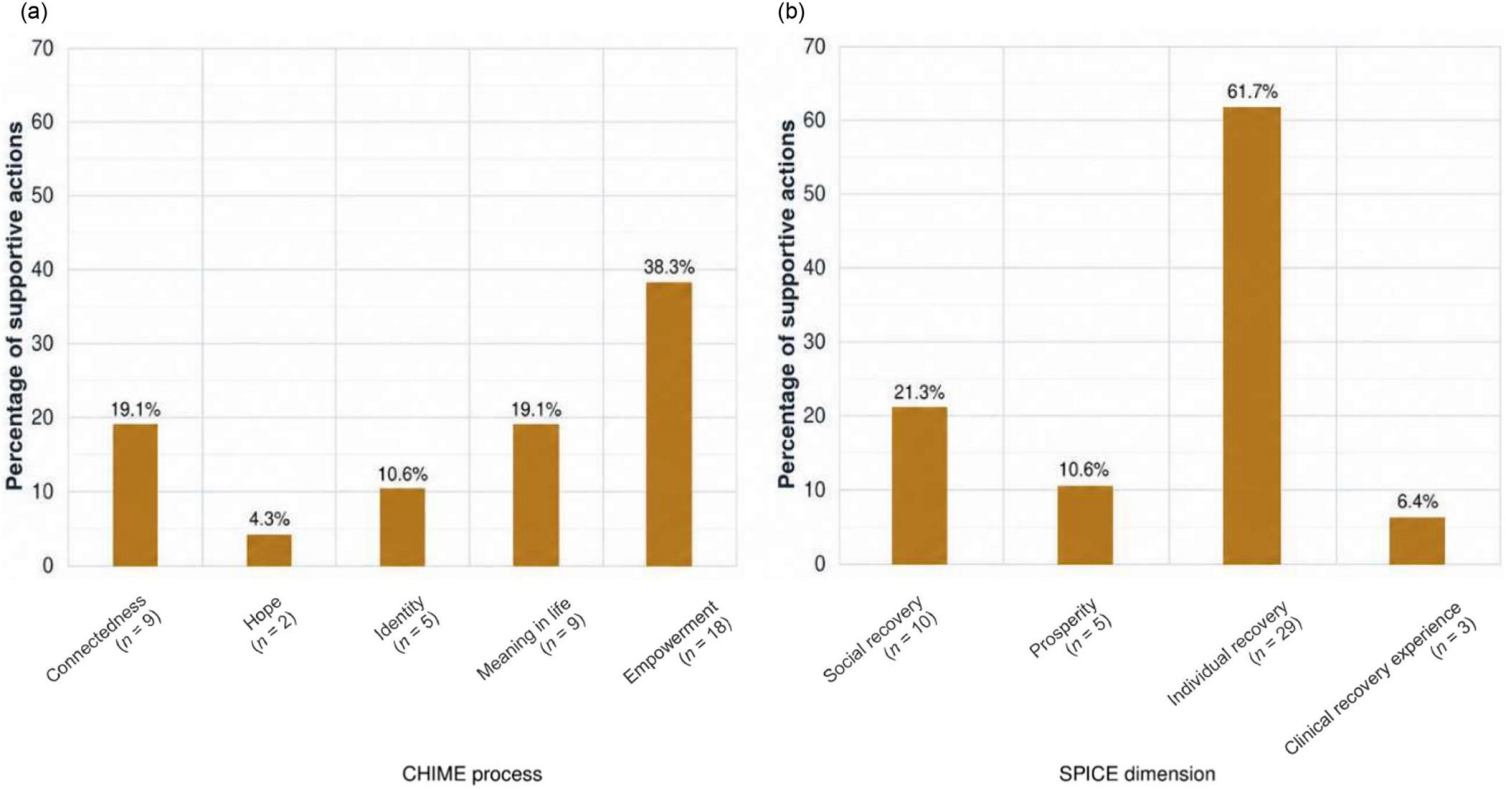
How the 47 *supportive actions* proposed by family caregivers aligned with (a) CHIME and (b) SPICE. *Note*. Each *supportive action* could align with one or more (a) CHIME processes and (b) SPICE dimensions. Nine *supportive actions* (19.1%) were not classified under any CHIME process, whereas all *supportive actions* were classified under at least one SPICE dimension. Percentages for the CHIME and SPICE classifications, together with the percentage of *supportive actions* not classified under any CHIME process, may sum to more than 100% because a *supportive action* could align with more than one CHIME process or SPICE dimension.

Figure 3 shows how family caregivers' *supportive actions* aligned with CHIME and SPICE. Tables S3 and S5 present, respectively, *supportive actions* that converged with statements on the *meaning of recovery and progress recognition* and those without direct convergence, together with their classifications under CHIME processes and SPICE dimensions.

## 4 Discussion

This paper reports the findings of two sequential studies conducted to explore recovery in mental health from the perspectives of key stakeholders: experts by experience, recovery‐oriented professionals and family caregivers. Study 1 examined convergences and unique contributions in how experts by experience and recovery‐oriented professionals understood the *meaning of recovery and progress recognition* and identified *facilitators and obstacles*. Study 2 investigated how family caregivers' *supportive actions* converged with the statements on the *meaning of recovery and progress recognition* identified in Study 1. In both studies, we examined how the identified statements and supportive actions were classified within the CHIME and SPICE frameworks. The findings provide valuable insights into shared and divergent views, highlighting diversity in how recovery is conceived by different stakeholder groups and the need to take into account a range of perspectives in recovery‐oriented practices and policies.

### 4.1 Meaning of Recovery and Progress Recognition

Most of the statements reflecting the *meaning of recovery and progress recognition* showed convergence across experts by experience and recovery‐oriented professionals, indicating substantial common ground in how recovery in mental health is understood. Both these stakeholder groups view recovery as a process of rebuilding after a mental health crisis so as to lead a fulfilling and meaningful life, which implies agency, empowerment, self‐awareness, self‐determination, autonomy and recognition as a subject with rights. They also understood recovery as coping with daily life and mental health challenges, overcoming self‐stigma and feeling good about oneself, supported by hope, safety, acceptance, connection, social relationships and leisure.

Experts by experience provided perspectives not captured by recovery‐oriented professionals, emphasising relational recovery (e.g., social support and interpersonal relationships), everyday well‐being (e.g., healthy lifestyles), socio‐economic security (e.g., having basic financial needs covered), rights‐based recovery (e.g., defending collective rights and legal capacity), and a critical reorientation away from the dominant biomedical model, framing recovery as an active pursuit of well‐being rather than merely symptom reduction [8, 9, 10]. These perspectives highlight essential aspects of recovery that professionals need to recognise in order to better understand service users' recovery processes. No statements concerning the *meaning of recovery and progress recognition* emerged exclusively from professionals, highlighting the irreplaceable role of lived experience in expanding traditional definitions [8, 12, 35].

Although most statements concerning the *meaning of recovery and progress recognition* were classified under one or more CHIME processes, several statements extended beyond its scope by foregrounding structural and rights‐based conditions for recovery that are not explicitly addressed within this framework. The CHIME processes most prominently represented were Empowerment and Meaning, whereas Hope was less evident. The relative under‐representation of Hope in our results is consistent with cautions against overly optimistic interpretations of recovery [25].

Importantly, CHIME does not explicitly address justice‐related statements concerning the *meaning of recovery and progress recognition*. These statements reflect calls to move away from the biomedical model of care due to the social and epistemic injustices that service users have reported in relation to biomedical approaches, including experiences of coercion, marginalisation and the devaluation of experiential knowledge [14, 36]. The present findings reveal an orientation towards collective recovery and human rights, consistent with previous publications [14, 37]. Experts by experience described recovery not only as personal well‐being but also as collective action, activism, and resistance to psychiatric violence and social injustice [1, 14], highlighting recovery as an inherently political process that links individual well‐being to broader struggles for rights and justice [14, 38]. This is consistent with previous critiques of CHIME regarding its limited attention to social and contextual factors [7, 21]. Together, these statements point to a recovery process centred on justice, understood as freedom from coercion, recognition of rights, material security and protection from harm. The inclusion of justice is particularly warranted, as recovery ‘is, at its heart, a social justice movement’ [39, p. 84].

While CHIME has been widely used to guide recovery‐oriented practice and service transformation [17], and plays a key role in challenging biomedical practices where coercion and rights restriction have been reported [14], relying on CHIME alone may risk overlooking vulnerability and justice‐related aspects of recovery, leading to overly individualistic or overly optimistic interpretations [40, 41]. Therefore, aspects of recovery not directly represented within CHIME should be considered alongside the framework to provide a more comprehensive understanding of recovery.

All statements concerning the *meaning of recovery and progress recognition* were classified under at least one SPICE dimension. This suggests that SPICE captured a broader range of the recovery meanings identified by stakeholders, particularly those related to social, economic and rights‐based conditions. In contrast to CHIME, none of the *meaning of recovery and progress recognition* statements fell outside the scope of SPICE. In particular, statements that were not classified under any CHIME process (e.g., basic financial needs, collective rights, safety or moving away from the biomedical model) were meaningfully captured within SPICE dimensions, including Prosperity, Social recovery, Individual recovery, and Clinical recovery experience [5].

Additionally, a substantial number of statements were classified under the Individual recovery dimension, consistent with the relevance of person‐centred experiences in recovery and supporting the continued relevance of personal recovery frameworks such as CHIME in guiding recovery‐oriented care [17]. However, the Individual recovery dimension encompasses heterogeneous elements (i.e., normalcy, individual responsibility, identity, knowledge, appearance and hygiene), some of which depend not only on personal agency but also on social norms, opportunities and material conditions (e.g., ‘achieving the desired quality of life’). This reflects a structural tension within the personal recovery frameworks on which SPICE is partly based (e.g., CHIME and SAMHSA), where experiences framed as ‘individual’ are nonetheless co‐produced through social, relational and material conditions [42]. Greater conceptual clarity may therefore be achieved by introducing distinctions within Individual recovery, similar to those proposed within Social recovery (i.e., internally and externally derived) [5]. This ambiguity has been widely discussed in the literature and highlights ongoing tensions between recovery as individual or social [9, 15], as well as calls for greater conceptual clarity to better account for shared responsibility and control in recovery processes [21].

Clinical recovery experience was minimally represented among statements concerning the *meaning of recovery and progress recognition*. In SPICE, this dimension refers to how diagnosis and treatment are experienced and to their implications for everyday life, including whether they are accepted, negotiated, contested or rejected [5]. The statement classified under this dimension described moving away from the dominant biomedical model by reducing emphasis on diagnosis and clinical input, rejecting coercive practices, and overcoming adverse treatment effects. It therefore reflected a critical position regarding diagnosis and treatment rather than clinical recovery goals or symptom remission.

While clinical interventions remain important for some service users [29, 40, 43], these accounts point to a transition away from a narrow biomedical understanding of clinical recovery towards a more rights‐based and experiential interpretation [1]. From this perspective, SPICE provides a useful framework for examining clinical recovery insofar as it allows this dimension to be approached critically, holistically and inclusively, incorporating service users' experiences and perspectives.

CHIME and SPICE differ in scope and conceptual structure. CHIME describes personal recovery processes, whereas SPICE offers a broader dimensional framework. Importantly, SPICE's Individual recovery and Social recovery dimensions are informed by personal recovery frameworks such as CHIME. The prominence of both dimensions therefore highlights the continued relevance of personal recovery. CHIME may remain appropriate for examining personal recovery processes, while SPICE may be useful when a broader analysis of social, material, rights‐based, and clinical experiences is required. The frameworks should therefore be understood as complementary.

### 4.2 Facilitators and Obstacles

In line with previous studies, experts by experience and recovery‐oriented professionals emphasised the importance of social and contextual conditions (such as safety, financial stability and social support), illustrating that recovery cannot be separated from material, relational and structural realities [25].

Unique contributions also emerged. Professionals emphasised peer support and community‐based care, reflecting the influence of theoretical perspectives in shaping recovery‐related knowledge [10]. Experts by experience, however, provided unique contributions not captured by professionals, emphasising ethically grounded and non‐coercive care, rights and protection from psychiatric violence. These findings highlight the value of co‐producing mental health policies, research and frameworks through genuine collaboration [44, 45], paying attention to power dynamics so as to ensure that lived experience is meaningfully incorporated and not overshadowed by professionalized knowledge [46, 47].

Our findings show that social and structural determinants—such as housing, financial resources and discrimination—were not classified under any CHIME process but were captured within the SPICE framework. Both convergent statements and unique contributions concerning *facilitators and obstacles* were represented within SPICE, particularly through its Social recovery and Prosperity dimensions. This pattern suggests that SPICE may be particularly useful for examining *facilitators and obstacles* from a broader perspective, given its explicit integration of structural and social factors through its Social Recovery and Prosperity dimensions, and its emphasis on the interplay between personal and structural factors as central to recovery [48].

The findings further highlight the need to consider structural disadvantages, such as poverty, discrimination and lack of safety [49, 50], supporting growing calls to integrate social justice, human rights and structural equity into recovery research [5, 21, 25, 49, 51].

### 4.3 Supporting Recovery

Most of the *supportive actions* proposed by family caregivers converged with the statements concerning the *meaning of recovery and progress recognition* identified in Study 1, indicating close correspondence between family caregiving practices and key elements of recovery. This likely reflects experiential and intuitive knowledge developed through sustained caregiving. For example, respecting service users' decisions and facilitating social engagement suggest an intuitive grasp of empowerment and relational support.

A smaller set of *supportive actions* emphasised family caregivers' own well‐being, such as self‐care and emotional management. These actions, which were identified as having no direct convergence with any statement concerning the *meaning of recovery and progress recognition*, highlight the dual role of families: supporting the service user while safeguarding their own wellness [52, 53]. This distinction underscores the need to differentiate between actions that foster user recovery and those that sustain caregiver well‐being, ensuring both are adequately addressed in practice, particularly in light of the challenges families face in their supportive role [3, 54].

Family caregivers' *supportive actions* did not explicitly address several statements concerning the *meaning of recovery and progress recognition*, particularly those related to empowerment, personal autonomy, identity, meaning in life, leisure, social acceptance, personal growth and the defence of collective rights. This pattern suggests the importance of clearer guidance to help families recognise how, and to what extent, they can contribute to these aspects of recovery, as well as how to navigate the limits of their role when such processes primarily depend on the service user's own agency or broader social conditions. Clarifying these boundaries may help families support recovery while avoiding over‐responsibility or unintended constraints on autonomy.

When examined in relation to CHIME, most *supportive actions* were classified under Empowerment and Connectedness, reflecting families' promotion of autonomy, advocacy, decision‐making and social inclusion. Conversely, only a small number of *supportive actions* were classified under the CHIME process of Hope, suggesting families may prioritise practical and relational support over explicitly nurturing optimism.

*Supportive actions* addressing conditions beyond intrapersonal recovery, such as financial support or monitoring treatment adverse effects, were not classified under any CHIME process. This extends understanding by showing that family support is not confined to intrapersonal recovery processes, but may also target conditions that shape whether support is possible. In line with Langford et al. [55], some of these can be understood as antecedents of social support, that is, the social, relational and material conditions (e.g., social networks, social embeddedness and social climate) that enable supportive relationships to occur.

All *supportive actions* were classified under at least one SPICE dimension, showing that each action corresponded to at least one of its dimensions. Most were classified under the Individual recovery dimension, reflecting families' involvement in diverse forms of practical, emotional and relational support. By contrast, relatively few *supportive actions* were classified under the Clinical recovery experience dimension, as these mainly concerned aspects of recovery more commonly addressed within professional or service‐led contexts, such as medication monitoring or seeking professional guidance.

More broadly, the contribution of *supportive actions* depends on how they are enacted. For example, the *supportive action* ‘clarifying the responsibilities of those involved in the recovery process’ may foster self‐determination when grounded in dialogue and negotiated responsibility, but could undermine recovery if enacted through unidirectional instruction or control. *Supportive actions* were considered compatible with recovery‐oriented care only when interpreted within a collaborative, informed and non‐coercive relationship with services, consistent with rights‐based and recovery‐oriented principles [14, 36].

Overall, these findings highlight the pivotal role that families play in enacting recovery‐oriented care, particularly due to their potential in fostering Empowerment and Connectedness. They also underscore the importance of recognising caregiver well‐being and providing targeted training to support families within person‐centred, rights‐based care [3, 56].

### 4.4 Strengths and Limitations

The present analysis has two main limitations. First, although efforts were made (in the primary studies) to include diverse voices, perspectives from underrepresented groups in mental health research—particularly from low‐ and middle‐income countries—may not have been fully captured, as European stakeholders predominated. Nonetheless, cross‐continent representation was secured. In addition, most family caregiver participants were members of family associations, representing a specific and organised subset of family caregivers. Consequently, the findings of Study 2 should be interpreted with caution and may not be generalisable to all family caregivers. Second, the use of ChatGPT in qualitative analysis, while efficient, remains at an early stage of methodological development. AI cannot fully capture contextual and interpretive nuances, may reproduce biases, and its outputs vary depending on prompts and system versions [57]. To mitigate these risks—and recognising that qualitative analysis is inherently subjective [58], requiring reflexivity and deep engagement with the data—we assessed human–AI agreement using Cohen's kappa in Study 1. The very good agreement observed supported the subsequent use of AI‐assisted procedures in Study 2. Furthermore, all AI‐assisted outputs were treated as provisional analytical proposals and critically reviewed by the research team.

Despite these limitations, this research provides the first evidence of convergence and unique contributions between the consensus perspectives of experts by experience and recovery‐oriented professionals, and of direct convergence and no direct convergence between family caregivers' *supportive actions* and statements concerning the *meaning of recovery and progress recognition*, examined in relation to two evidence‐based frameworks (i.e., CHIME and SPICE). A key strength lies in its focus on the differentiated contributions of each stakeholder group to understanding recovery. By systematically comparing these perspectives, the analysis identifies areas of both convergence and unique contributions, offering actionable insights to promote person‐centred recovery within each group. Understanding what each group values— and which aspects are emphasised differently across perspectives—enables more nuanced interventions and policies that respect these varied priorities. Additionally, the use of data from three e‐Delphi studies ensures a diverse and well‐informed participant base. Finally, the incorporation of AI‐assisted analytical procedures using a large language model (i.e., ChatGPT) represents an innovative methodological contribution, demonstrating how AI can be critically integrated into qualitative analysis.

### 4.5 Future Research

Future studies should investigate relationships between CHIME processes and SPICE dimensions across diverse cultural contexts so as to provide a more comprehensive account of recovery—particularly among minority groups and in low‐ and middle‐income countries. Greater attention should also be paid to the specific roles and needs of family caregivers. Research designs must actively engage service users, families and communities—with transparent reporting of Patient and Public Involvement (PPI)—to foreground lived experience and counterbalance biases and power imbalances rooted in biomedical traditions. The feasibility of developing a comprehensive recovery‐oriented model to guide mental health services—rather than relying on the biomedical model—should likewise be explored. Such a model should be grounded in the original philosophy of the recovery movement, clarify existing conceptual ambiguities, and be guided by its foundational principles in both theory and practice. Finally, further research is needed to enhance the assessment of recovery and its key facilitators and obstacles, thereby improving routine outcome evaluation. In addition, studies should examine whether the recovery process may have an endpoint for some individuals, ensuring that recovery does not become a new pathway to chronicity in which people lose the right to decide when their own recovery concludes, regardless of symptom persistence.

## 5 Conclusion

This article advances understanding of mental health recovery by synthesising the perspectives of experts by experience, recovery‐oriented professionals and family caregivers across two complementary studies. Although recovery is understood and experienced in individual and context‐dependent ways, the findings identify convergences between experts by experience and recovery‐oriented professionals and unique contributions from each group, while showing that most family caregivers' *supportive actions* directly converged with statements concerning the *meaning of recovery and progress recognition*.

Experts by experience made unique contributions concerning rights, everyday well‐being and a critical stance towards coercive and narrowly biomedical approaches, whereas recovery‐oriented professionals emphasised occupational opportunities, autonomy, self‐awareness, peer support and community‐based care. Most family caregivers' supportive actions directly converged with statements concerning the meaning of recovery and progress recognition, while actions with no direct convergence highlighted caregivers' own capacities and well‐being.

The findings also demonstrate the complementary value of the CHIME and SPICE frameworks. CHIME provides a valuable framework for understanding personal recovery processes, whereas SPICE captured additional social, material, rights‐related and clinical aspects, including content not classified under CHIME. The two frameworks captured overlapping yet complementary aspects and, when used together, may provide a more comprehensive understanding of recovery.

Overall, these findings support a multidimensional and rights‐based conception of recovery that integrates personal, relational and structural dimensions. They also highlight the importance of recognising the unique contributions of experts by experience and recovery‐oriented professionals, together with family caregivers' supportive actions when developing recovery‐oriented policies, services and interventions. Despite differences across stakeholder perspectives, the convergences identified provide a foundation for advancing recovery‐oriented mental health care, future research and service development.

### 5.1 Relevance for Clinical Practice

This paper offers actionable insights for advancing recovery‐oriented, person‐centred mental health care. By grounding the analysis in lived experience, applying the CHIME and SPICE frameworks, and comparing the perspectives of experts by experience, recovery‐oriented professionals and family caregivers, the findings emphasise the importance of holistic care planning and recognising family caregivers as important contributors to recovery. In making explicit the convergences between experts by experience and recovery‐oriented professionals, the unique contributions of each group, and family caregivers' supportive actions, the research can support greater mutual understanding, facilitate dialogue, and inform more collaborative and responsive clinical practice. These insights may guide service development, assessment and intervention design, ultimately contributing to a more equitable, compassionate and effective mental health system.

## Author Contributions

**Estefania Guerrero:** conceptualization, data curation, formal analysis, investigation, methodology, software, writing – original draft, writing – review and editing, validation, visualization. **Maite Barrios:** conceptualization, investigation, methodology, supervision, writing – review and editing, validation. **Hernán Maria Sampietro:** conceptualization, formal analysis, investigation, writing – review and editing, validation. **Juana Gómez‐Benito:** funding acquisition, methodology, project administration, resources, supervision, validation. **Georgina Guilera:** conceptualization, formal analysis, funding acquisition, investigation, methodology, project administration, resources, supervision, writing – review and editing, validation.

## Ethics Statement

Data in this study did not contain any user‐identifiable information. As such, ethical approval was not required; however, the research was conducted in accordance with the ethical principles of the Declaration of Helsinki (World Medical Association, 1975; most recent revision 2013) and the University of Barcelona's Code of Research Integrity.

## Conflicts of Interest

The authors declare no conflicts of interest.

## Use of Artificial Intelligence (AI) Tools

Artificial intelligence (AI) tools were used to support specific stages of the analytical and writing processes. ChatGPT (version 4o; web interface) generated provisional classifications of statements into CHIME processes and SPICE dimensions in Study 1. In Study 2, it generated provisional proposals concerning conceptual convergence, together with classifications of supportive actions into CHIME processes and SPICE dimensions. During manuscript preparation, the tool was also employed to refine wording in the initial draft. All AI‐generated outputs were treated as provisional and were critically reviewed and edited by the authors. The final manuscript underwent professional language editing.

## Supporting information


Supporting File

## Data Availability

All data analysed in this study are open access and available in the original publications cited in the manuscript.
