# Supplementary material for: Recovery in Mental Health: A Synthesis of the Perspectives of Experts by Experience, Recovery‐Oriented Professionals and Family Caregivers in Relation to the CHIME and SPICE Frameworks
Source: Health Expect. 2026 Aug 19;29(4):e70815. doi: 10.1111/hex.70815 (PMC13488006; doi:10.1111/hex.70815)
Supplement: Supplementary file 1 [file HEX-29-e70815-s001.docx]

**SUPPLEMENTARY MATERIAL**

- Table S1. Participant recruitment and inclusion criteria
- Table S2. ChatGPT prompts used to support AI-assisted analytical procedures in Study 1 and Study 2
- Table S3. Convergences between and unique contributions of integrated statements on the *meaning of recovery and progress recognition* and *supportive actions*, showing how the statements and *supportive actions* aligned with CHIME and SPICE
- Table S4. Convergences between and unique contributions of integrated statements concerning *facilitators and obstacles*, showing how the statements aligned with CHIME and SPICE
- Table S5. *Supportive actions* that did not converge with the statements on the *meaning of recovery and progress recognition*, showing how they aligned with CHIME and SPICE

**Table S1.** Participant recruitment and inclusion criteria

| **Delphi** | **Stakeholders** | **Inclusion criteria** | **Recruitment strategies** |
| --- | --- | --- | --- |
| 1 (authors1) | Experts by experience | (a) Over 18 years of age  (b) Identifies as a user of mental health services or survivor of psychiatry  (c) Either a member of a users/survivors of psychiatry association or an author of technical/scientific publications based on lived experience | (1) Contacting associations of users and survivors of psychiatry and affiliated members (convenience sampling)  (2) Identifying authors of relevant publications (convenience sampling)  (3) Reaching out to QualityRights contributors  (4) Snowball sampling through participant networks |
| 2  (authors2) | Professionals | (a) Recovery-oriented professionals (e.g. nurses, occupational therapists, psychiatrists, psychologists)  (b) Minimum two years' experience in recovery-oriented services or significant field contributions  (c) Working with users over 18 | (1) Searching electronic databases and publications (convenience sampling)  (2) Contacting QualityRights contributors (convenience sampling)  (3) Expert referrals from previous steps (snowball sampling) |
| 3  (authors3) | Family caregivers | (a) Self-identify as family members supporting users of mental health services  (b) Support users over 18 years of age | (1) Gathering contacts from global family association websites (convenience sampling)  (2) Encouraging participants to share invitations within their networks (snowball sampling) |

**Table S2.** ChatGPT prompts used to support AI-assisted analytical procedures in Study 1 and Study 2

| **a) ChatGPT prompt used in Study 1 to align statements with CHIME and SPICE: Example for statements concerning *facilitators and obstacles*** | **b) ChatGPT prompt used in Study 2 to generate provisional proposals concerning conceptual convergence and provisional CHIME/SPICE classifications** |
| --- | --- |
| I will now provide you with statements about facilitators and barriers in the recovery process. Please read each statement carefully and align it with the relevant CHIME processes and SPICE dimensions, providing a brief justification for your choice. You may use “All” if the statement applies to all processes or dimensions, or “None” if it does not apply to any | You will receive two sets of statements:  Set 1: A group of 33 statements describing the conceptual core of recovery in mental health. Each statement reflects key aspects that define recovery or indicate that recovery is taking place. These statements are aligned with the established recovery frameworks such as CHIME (Connectedness, Hope, Identity, Meaning, Empowerment) and SPICE (Social recovery, Prosperity, Individual recovery, Clinical recovery experience).  Set 2: A list of supportive actions that have been identified by family members as the most relevant and helpful actions to support a person’s recovery.  Your task is as follows:  Converge each supportive action from Set 2 with the most conceptually aligned statement from Set 1.  The convergence should be based on what the supportive action is clearly promoting in terms of the conceptual core of recovery.  Provide a brief justification for each convergence, explaining why the supportive action converges with that specific aspect of recovery.  For any supportive action in Set 2 that does not clearly converge with a statement from Set 1, list it separately.  Provide a justification for why no direct convergence could be made.  Then, assess whether that action may still align with the CHIME and/or SPICE frameworks.  If applicable, identify which CHIME process or SPICE dimension it best corresponds to and explain your reasoning.  Your output should include:  The matched pairs (Supportive Action → Recovery Statement) with justifications.  A separate section for unmatched Supportive Actions, each with:  a) An explanation of why it doesn’t converge with any of the 33 core recovery statements.  b) A proposed alignment with a CHIME process and/or SPICE dimension, if applicable, and a justification.  Maintain clarity, consistency and conceptual fidelity throughout. |

*Note.* The prompts are reproduced verbatim as used during the analysis. In the prompts, “conceptual core” refers to the Study 1 statements on the *meaning of recovery and progress recognition*, and “barriers” corresponds to the term “obstacles” used in the manuscript.

**Table S3.** Convergences between and unique contributions of integrated statements on the *meaning of recovery and progress recognition* and *supportive actions*, showing how the statements and *supportive actions* aligned with CHIME and SPICE

| **Meaning of recovery and progress recognition** | **CHIME** | | | | | **SPICE** | | | | **Supportive actions** |
| --- | --- | --- | --- | --- | --- | --- | --- | --- | --- | --- |
|  | **C** | **H** | **I** | **M** | **E** | **S** | **P** | **Ir** | **CE** |  |
| Achieving the desired quality of life (e.g. experiencing well-being, life satisfaction, and a general sense of accomplishment). |  |  |  | x |  |  |  | x |  | Focusing on good, beautiful, quiet things (e.g. nature). |
| **Being able to enjoy one’s own life (e.g. enjoying hobbies, being happy in society, and having a sense of humour).** |  |  |  | x |  |  |  | x |  | Sharing enjoyable activities with the user (e.g. walking, doing sports, gardening, manual and creative work). |
| **Being assertive (e.g. establishing healthy boundaries with people, and asserting one’s own opinion).** |  |  |  |  | x |  |  | x |  | Explaining our own expectations and the difficulties that arise in the process of accompanying the user. |
| Being recognized as a subject with rights (e.g. having one's limits and decisions respected, making informed choices about treatment and recovery through advance directives). |  |  |  |  | x |  | x |  |  | Being able to advocate on behalf of the user in front of professionals if needed.  Building trust with the user, making clear that their rights will not be violated (i.e. treating the user as an equal, seeing the person and not the illness). |
| Coping with the demands of daily life (e.g. addressing one’s own needs). |  |  |  |  | x |  |  | x |  | Being able to ask the user if they need help and how to help them. |
| Dealing with mental health problems (e.g. asking for help, preventing relapse, coping with conflicts, symptoms, and other challenges along the recovery path). |  |  |  |  | x |  |  | x |  | Accepting the user's condition (e.g. considering the condition as a lifelong process).  Appreciating and supporting the efforts and progress of the user (e.g. attempts to cope with the illness or to improve behaviours).  Encouraging the user to diligently take prescribed medicines as advised.  Helping the user to keep up with appointments with psychiatrists, therapists and other healthcare professionals.  Understanding the illness, symptoms and distress of the user. |
| **Defending collective rights (e.g. against psychiatric violence or social barriers, with awareness of one's legal capacity—the right to have rights and to exercise them).** |  |  |  |  |  |  | x |  |  |  |
| Developing a sense of agency (i.e. actively making meaningful decisions and exerting independent capability to act on one's own will). |  |  |  |  | x |  |  | x |  | Trusting the user. |
| Developing empowerment (i.e. regaining control of one’s life and making autonomous decisions). |  |  |  |  | x |  |  | x |  |  |
| Enjoying leisure and free time (e.g. engaging in activities one enjoys). |  |  |  | x |  |  |  | x |  |  |
| Feeling accepted and valued by others. | x |  |  |  |  | x |  |  |  |  |
| Feeling connected to others (e.g. experiencing a sense of belonging and relationship). | x |  |  |  |  | x |  |  |  | Encouraging networking with user groups in the community (i.e. mutual support).  Maintaining the bond with the user (i.e. a good understanding). |
| Feeling good about oneself (e.g. having a positive identity, self-esteem, self-concept, and confidence in one’s capabilities). |  |  | x |  |  |  |  | x |  |  |
| Feeling safe (e.g. having the necessary conditions to live securely). |  |  |  |  |  |  | x |  |  | Accompanying the user to take advantage of available services. |
| Feeling there is meaning in life. |  |  |  | x |  |  |  | x |  |  |
| **Getting a personal meaningful purpose out of one’s own experience (e.g. making sense of one’s experience and achieving self-acceptance).** |  |  |  | x |  |  |  | x |  | Giving time and space for the user to talk about their experiences without the fear of judgment. |
| Growing as an individual on different levels (i.e. thriving by learning to live and enjoy oneself despite diagnosis or symptoms, and developing resilience through a healthy lifestyle and managing personal suffering and stress). |  |  |  | x | x |  |  | x |  |  |
| **Having a healthy lifestyle (e.g. eating well, exercising, good sleep hygiene, does not abuse substances).** |  |  |  |  |  |  |  | x |  | Encouraging the user to do things that could help to sustain recovery (e.g. exercise, adequate nutrition). |
| **Having a personal path and life goal (e.g. achieving goals or progressing toward them).** |  |  |  | x |  |  |  | x |  | Helping the user to find what might interest them (e.g. proposing activities without insisting).  Helping to set up clear goals for the user (e.g. social life, work, independent living).  Understanding there is no single or "right" path to recovery. |
| Having a satisfactory social life (e.g. feeling accepted by others and enjoying relationships). | x |  |  |  |  | x |  |  |  | Supporting social inclusion of the user (e.g. family, friends, work). |
| **Having basic financial needs covered.** |  |  |  |  |  |  | x |  |  | Having sufficient financial resources. |
| Having hope (i.e. being hopeful about recovery, the future, or achieving personal goals). |  | x |  |  |  |  |  | x |  | Having a positive attitude (e.g. showing resilience, being optimistic, having patience, being empathic).  Having hope in recovery. |
| Having personal autonomy (i.e. independent living and depending less on professionals and family members). |  |  |  |  | x |  |  | x |  |  |
| Having self-awareness (i.e. recognizing and assuming one's capacities, difficulties, strengths, weaknesses, needs, preferences, progress, and achievements). |  |  | x |  | x |  |  | x |  | Assisting the user in expressing needs in the recovery process.  Knowing the needs of the user.  Listening to the user (e.g. listening to their concerns, needs and expectations). |
| Having self-determination (i.e. being the protagonist of one’s own process by making decisions about one’s identity, future, goals, needs, activities, and treatment without control from others). |  |  |  |  | x |  |  | x |  | Clarifying the responsibilities of those involved in the recovery process.  Having respect and consideration for the user (e.g. respecting the user's decisions, not giving unsolicited advice, not coercing).  Participating in decision-making and treatment plans. |
| **Having support from significant others (e.g. a supportive social network including family and friends).** | x |  |  |  |  | x |  |  |  | Being present and proactive.  Giving love and affection to the user.  Providing the necessary support to the user (e.g. emotional support). |
| **Improving interpersonal relationships.** | x |  |  |  |  | x |  |  |  | Communicating honestly, respectfully and clearly (e.g. knowing how to de-escalate conversations). |
| **Improving wellness (e.g. reducing distress and managing personal suffering and stress).** |  |  |  |  | x |  |  | x |  | Being aware of trigger factors and red flags. |
| **Knowing helpful recovery strategies.** |  |  |  |  | x |  |  | x |  | Learning about the recovery process and the available alternatives (e.g. health and social services, resources in the community). |
| Leading a fulfilling life (e.g. living meaningfully according to one’s major goals). |  |  |  | x |  |  |  | x |  | Supporting activities that the user wants to do. |
| **Moving away from the dominant biomedical model approach (i.e. reducing emphasis on diagnosis, clinical input, involuntary detention, and forced treatment, while overcoming the adverse effects of psychiatric drugs and treatments).** |  |  |  |  |  |  |  |  | x | Being aware of the effects of medication.  Trusting in healthcare professionals and asking for professional advice. |
| Overcoming self-stigma (e.g. constructing a personal and social identity beyond the mental health problem and leaving the sick role). |  |  | x |  |  |  |  | x |  | Contributing to eliminating stigma in society.  Eliminating our own stigma towards the user. |
| Rebuilding one’s life after a mental health crisis (e.g. resuming relationships and employment, engaging in new activities, and becoming an active member of the community through participation and a sense of belonging). | x |  |  | x |  | x |  |  |  | Providing access to appropriate professionals and treatments.  Strengthening the reintegration process in the user's ordinary environment. |

*Note.* C: Connectedness; H: Hope; I: Identity; M: Meaning; E: Empowerment; S: Social recovery; P: Prosperity (i.e. political and economic recovery); Ir: Individual recovery; CE: Clinical recovery experience. Unique contributions of experts by experience are shown in bold.

**Table S4.** Convergences between and unique contributions of integrated statements concerning *facilitators and obstacles*, showing how the statements aligned with CHIME and SPICE

| **Facilitators and obstacles** | **CHIME** | | | | | **SPICE** | | | |
| --- | --- | --- | --- | --- | --- | --- | --- | --- | --- |
|  | **C** | **H** | **I** | **M** | **E** | **S** | **P** | **Ir** | **CE** |
| Availability in the living environment of basic services (e.g. transport, water, electricity, etc.). |  |  |  |  |  |  | x |  |  |
| Being able to act with self-determination (e.g. opportunities to make one's own decisions). |  |  |  |  | x |  |  | x |  |
| Being subjected to coercion (e.g. other people interfere so that my decisions suit their interests or preferences rather than mine). |  |  |  |  | x | x | x |  |  |
| *Contact with professionals who promote self-awareness (e.g. about strengths, weaknesses, needs, preferences, etc.).* |  |  | x |  | x |  |  | x |  |
| Financially secure enough to cover basic needs (e.g. housing and food). |  |  |  |  |  |  | x |  |  |
| *Gaining hope through contact with professionals and the community.* | x | x |  |  |  | x |  | x |  |
| *Having a meaningful and satisfying social life.* | x |  |  | x |  | x |  |  |  |
| Having a social environment characterized by support and respect (e.g. emotionally stable, safe and non-paternalistic). | x |  |  |  | x | x |  |  |  |
| *Having access to mental health care in one's own community (i.e. non-institutionalization).* | x |  |  |  | x | x | x |  |  |
| *Having adequate, regular, affordable, and easily accessible care services.* |  |  |  |  |  |  | x |  |  |
| Having meaning in life. |  |  |  | x |  |  |  | x |  |
| *Having occupational opportunities (e.g. to work, study, or volunteer).* |  |  |  | x |  | x |  | x |  |
| *Having opportunities to participate in meaningful activities.* |  |  |  | x |  | x |  | x |  |
| *Having personal autonomy (i.e. living independently).* |  |  |  |  | x |  | x | x |  |
| *Having psychological resources (e.g. self-esteem, coping strategies, etc.).* |  |  |  |  | x |  |  | x |  |
| *Having self-awareness (e.g. about one's own strengths, weaknesses, needs, preferences, etc.).* |  |  | x |  | x |  |  | x |  |
| Having the support of significant others (e.g. family, friends). | x |  |  |  |  | x |  |  |  |
| **Healthcare professionals act ethically (e.g. do not stigmatize, do not have conflicts of interest, are aware of their limitations).** | x |  |  |  | x | x | x |  |  |
| **Human rights are respected (e.g. the right to liberty, to equal recognition before the law)** |  |  |  |  |  |  | x |  |  |
| **Living in an inclusive and equitable society (e.g. one that is accepting of diversity).** |  |  |  |  |  | x | x |  |  |
| Not suffering discrimination based on gender, ethnicity or other attributes. |  |  |  |  |  |  | x |  |  |
| Not suffering social exclusion. | x |  |  |  |  | x |  |  |  |
| Overcoming self-stigma. |  |  | x |  |  |  |  | x |  |
| Overcoming social stigma |  |  | x |  |  | x |  |  |  |
| Overcoming the sick-role. |  |  | x |  |  |  |  | x |  |
| **Receiving care that is free from psychiatric violence (e.g. no forced treatments, involuntary admissions, etc.).** |  |  |  |  |  |  | x |  |  |
| *Receiving information about user rights (e.g. about advanced care planning or the recovery plan).* |  |  |  |  | x |  | x |  |  |
| *Receiving peer support.* | x |  |  |  |  | x |  |  |  |
| The living environment is safe. |  |  |  |  |  |  | x |  |  |
| Treatment approach is holistic, not only focused on symptoms. |  |  |  | x | x |  |  | x | x |

*Note.* C: Connectedness; H: Hope; I: Identity; M: Meaning; E: Empowerment; S: Social recovery; P: Prosperity (i.e. political and economic recovery); Ir: Individual recovery; CE: Clinical recovery experience. Unique contributions of experts by experience are shown in bold, and unique contributions of recovery-oriented professionals are shown in italics.

**Table S5.** *Supportive actions* that did not converge with the statements on the *meaning of recovery and progress recognition*, showing how they aligned with CHIME and SPICE

| **Supportive actions** | **CHIME** | | | | | **SPICE** | | | |
| --- | --- | --- | --- | --- | --- | --- | --- | --- | --- |
|  | **C** | **H** | **I** | **M** | **E** | **S** | **P** | **Ir** | **CE** |
| Having skills to deal with our own emotions and concerns. |  |  |  |  |  |  |  | x |  |
| Learning about mental health (e.g. attending colloquiums and talks). |  |  |  |  |  |  |  |  | x |
| Participating in an association (e.g. of families). |  |  |  |  |  | x |  |  |  |
| Practising self-care. |  |  |  |  |  |  | x |  |  |

*Note.* C: Connectedness; H: Hope; I: Identity; M: Meaning; E: Empowerment; S: Social recovery; P: Prosperity (i.e. political and economic recovery); Ir: Individual recovery; CE: Clinical recovery experience.
